# Effects of insect-specific viruses on transmission, infection, and replication of arboviruses in co-infected mosquitoes: a systematic review

**DOI:** 10.1186/s13071-026-07495-x

**Published:** 2026-06-23

**Authors:** Angela Sibanda-Makuvise, Nosipho Masoto, Gert Ignatius du Preez Terblanche, Puleng Ralepeli, Jolene Botha, Felicity J. Burt

**Affiliations:** 1 Division of Virology, Faculty of Health Sciences, University of the Free State, 205 Nelson Mandela Dr, Bloemfontein, 9301 South Africa; 2 Department of Applied Biology and Biochemistry, National University of Science & Technology, Cnr. Cecil Avenue/ Road, Ascot, Box AC 939, Bulawayo, Zimbabwe; 3 Division of Virology , National Health Laboratory Service, Universitas, Bloemfontein, 9301 South Africa

**Keywords:** Insect-specific viruses (ISVs), Insect-specific flaviviruses (ISFs), Arboviruses, Biocontrol, Vector competence, Replication inhibition, Transmission dynamics

## Abstract

**Background:**

Climate change, globalization, and urbanization continue to reshape the ecology of vector-borne diseases, allowing mosquito-transmitted arboviruses to expand into previously non-endemic areas. Increasing insecticide resistance, ecological disruption, and declining efficacy of conventional vector control strategies have prompted an interest in microbe-based alternatives. Insect-specific viruses (ISVs), a group of viruses restricted to replicating in arthropods like mosquitoes, have emerged as potential modulators of mosquito vector competence. This systematic review assesses current in vivo evidence on the effects of ISVs on arboviral replication, infection, and transmission, for the purpose of considering their potential application as biocontrol agents for arboviruses.Please confirm if the author names are presented accurately and in the correct sequence (given name, middle name/initial, family name).

**Methods:**

A systematic search strategy was applied to PubMed, Scopus, ScienceDirect, and Google Scholar to identify relevant studies. The selection process followed PRISMA guidelines and PICO-based inclusion criteria. RAYYAN AI was used to remove duplicates, for abstract and full text screening. Only in vivo studies analyzing interactions between ISVs and medically relevant arboviruses in mosquitoes were retained. Study quality was assessed using the Mixed Methods Appraisal Tool (MMAT), and data extraction followed JBI guidelines.

**Results:**

A total of 3386 studies were found using the search strategy, of these 15 studies met the inclusion criteria. The effects of ISVs on co-infecting arboviruses were highly variable, depending on mosquito species, ISV strain, route of infection, and the phylogenetic relationship between the ISV and the arbovirus. Cell-fusing agent virus (CFAV), Nhumirim virus (NHUV), Palm Creek virus (PCV), Culex flavivirus (CxFV), Eilat virus (EILV), Espirito Santo virus (ESV), Yichang virus (YCV), Anopheles gambiae densovirus (AgDNV), and Aedes aegypti densovirus (AaeDV) showed suppression in either one or more of the parameters of vector competence used, such as replication, transmission, and infection rate, likely associated with mechanisms consistent with superinfection exclusion or competition for shared cellular resources. In contrast, Phasi Charoen-like virus (PCLV) and Humaita-Tubiacanga virus (HTV) enhanced flavivirus infection and transmission in *Aedes aegypti,* suggesting that some ISVs may facilitate, rather than inhibit, arbovirus spread. Additionally, in some studies where infection routes differed, neutral or inconsistent interactions were observed.

**Conclusions:**

ISVs act as context-dependent modulators of arboviral dynamics in mosquitoes. While some indicate biological inhibitory potential, others enhance viral replication or have negligible effects, underlining the importance of meticulous viral selection and ecological risk assessment before considering ISVs as biocontrol candidates. Carefully designed experimental, mechanistic and semi-field evaluations are important for ISVs to be considered as biocontrol agents within integrated vector management programs.

**Graphical Abstract:**

**Figure:**
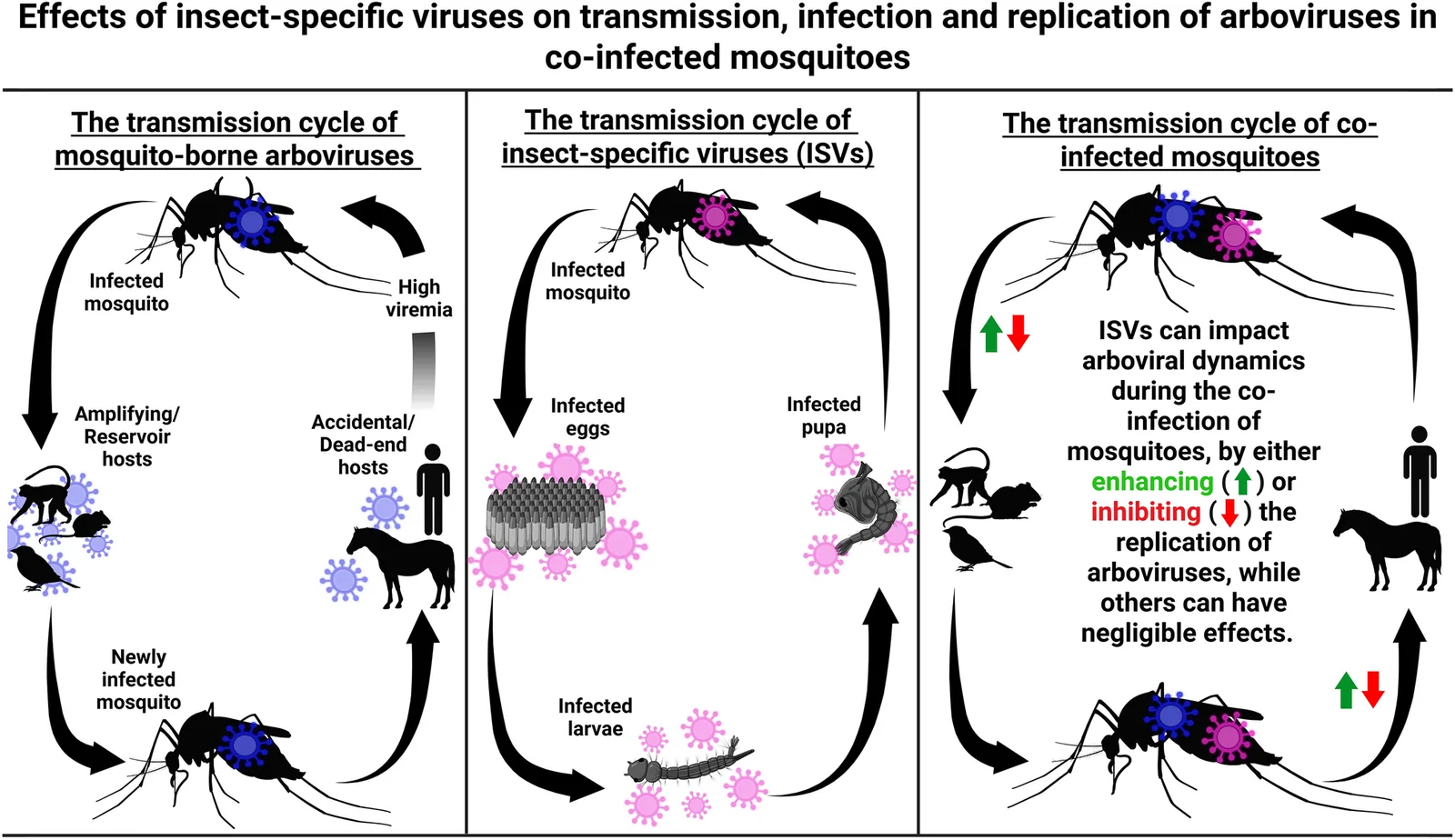

LE: This title is mismatch with jobsheet title. Please check if it is correct or not and proceed further.we have changed the picture title to match the manuscript title

**Supplementary Information:**

The online version contains supplementary material available at https://doi.org/10.1186/s13071-026-07495-x.

## Background

Among the vector-borne pathogens, arboviruses transmitted by mosquitoes and ticks are among the most important causes of global human and animal infectious disease. Arboviruses include viruses within the following families, the *Orthoflaviviridae* (genus Ortho*flavivirus*), *Togaviridae* (genus *Alphavirus*), *Phenuiviridae* (genus *Phlebovirus*), and the *Peribunyaviridae* (genus *Orthobunyavirus*). Over the past few decades, the global landscape of arbovirus transmission has changed significantly, with arboviruses being detected in previously non-endemic areas. For instance, autochthonous cases of dengue virus (DENV) and chikungunya virus (CHIKV) have been reported in Europe [1], and autochthonous cases of West Nile virus (WNV) were reported in the Netherlands [2, 3]. The same trend is seen with the mosquito host vectors. For instance, *Aedes albopictus*, originally found in Asia, has now spread to all continents, including Europe [4, 5], *Aedes aegypti* has recently been reported in Colorado, USA, and *Anopheles stephensi* is now increasing the risk of o’nyong-nyong virus transmission in urban areas in Africa [6]. In addition to environmental changes, demographical and social changes such as human travel, containerized shipping, urbanization, population growth, and changes in land use further contribute to the spread of arboviruses and their vectors [7, 8].

As a result, approximately 80% of the global population resides in areas at risk of infection with arboviruses, particularly those transmitted by mosquitoes [9]. DENV alone is responsible for approximately 400 million cases every year [10], while emerging arboviruses such as WNV, Zika virus (ZIKV), Usutu virus (USUV), Mayaro virus (MAYV), and CHIKV continue to present major public health concerns. Disturbingly, there are no specific targeted treatments and limited vaccines for arboviruses, leaving disease control to rely primarily on vector control and mosquito bite prevention methods. However, conventional approaches to mosquito control and mosquito bite prevention such as use of insecticides and repellents have raised environmental concerns, public health risks, increasing insecticide resistance, and behavioral adaptation of vectors [11]. In response, microbe-based strategies have gained traction as environmentally friendly alternatives for reducing infection and transmission of mosquito borne viruses [12, 13]. Notably, introduction of the bacterial endosymbiont *Wolbachia* into mosquito populations has significantly decreased dengue transmission in the field [14].

Naturally, mosquitoes contain diverse microorganisms that can affect the ability of mosquitoes to acquire and transmit arboviruses [15]. Among the microbiome of mosquitoes, there are insect-specific viruses (ISVs), that replicate exclusively in insects, maintained within the population through vertical and horizontal transmission and have no known pathogenicity in vertebrates [15]. ISVs belong to but are not restricted to the following viral families: *Flaviviridae*, *Phenuiviridae*, *Peribunyaviridae*, *Rhabdoviridae*, *Mesoniviridae*, *Parvoviridae*, *Togaviridae*, *Tymoviridae*, *Orthomyxoviridae*, *Birnaviridae*, *Nodaviridae*, *Reoviridae*, *Iridoviridae*, *Permutotetraviridae*, *Totiviridae,* and *Iflaviridae* [15]. Most known ISVs are classified within the family *Flaviviridae*, genus *Flavivirus*, and are collectively referred to as insect-specific flaviviruses (ISFs) [16]. Examples of ISFs include cell-fusing agent virus (CFAV), Nhumurim virus (NHUV), Palm Creek virus (PCV), and *Culex flavivirus* (CxFV).

Interestingly, ISVs have shown contradictory effects on replication, transmission, and infection of arboviruses in co-infected mosquitoes, with some ISVs demonstrating inhibitory and/or suppression of infection, while others enhance viral replication and transmission and others show no or negligible effects [17–30]. The aim of this study was to compile and analyze all the available information on the effects of ISVs on the replication, infection, and transmission of arboviruses in mosquitoes using a systematic review of published literature.

## Methods

For this review, we examined evidence of inhibition and/or suppression of replication of arboviruses, infection of mosquito cells by arboviruses, and transmission of arboviruses in the presence of ISV. The Preferred Reporting Items for Systematic Reviews and Meta-Analyses (PRISMA) guidelines were followed [31].

### Search strategy

A comprehensive evaluation of the literature was performed, using the scientific databases PubMed (https://pubmed.ncbi.nlm.nih.gov/ (accessed on 14 October 2025)), Scopus (https://www.elsevier.com/products/scopus/search (accessed on 14 October 2025)), ScienceDirect (https://www.sciencedirect.com/(accessed on 15 October 2025)) and Google Scholar (https://scholar.google.com/(accessed on 14 October 2025)) for peer-reviewed laboratory-based reports. Only English search keywords were used to retrieve literature from databases; insect-specific flavivir* OR ISF OR Insect-specific vir* OR ISV) AND (biocontrol OR replication OR transmission OR inhibition) AND arbovir* AND mosquit*. No restrictions were applied regarding the year of publication, allowing the inclusion of all available research. The complete Boolean search strings, database-specific search strategies, and applied filters are provided in Supplementary Sheet 1.

### Selection criteria

This study employed the Population, Intervention, Comparison, and Outcome (PICO) framework to guide the formulation of the review question and the development of inclusion criteria. The systematic review focused on mosquito vectors of arboviruses (Population), which are responsible for transmitting significant human diseases. The intervention of interest is the co-infection of mosquito vectors with ISVs and other arboviruses of medical significance, either naturally or experimentally introduced (Intervention). The comparison involved mosquitoes that are not co-infected with an ISV and an arbovirus (Comparison). The primary outcomes of interest were changes in the replication and transmission of arboviruses within mosquito vectors and the changes in arboviral infection and transmission cycles (Outcome).

**Review question:** Do ISVs affect transmission, infection, and replication of arboviruses in co-infected mosquitoes?

### Study selection and quality assessment

**Fig. 1:**
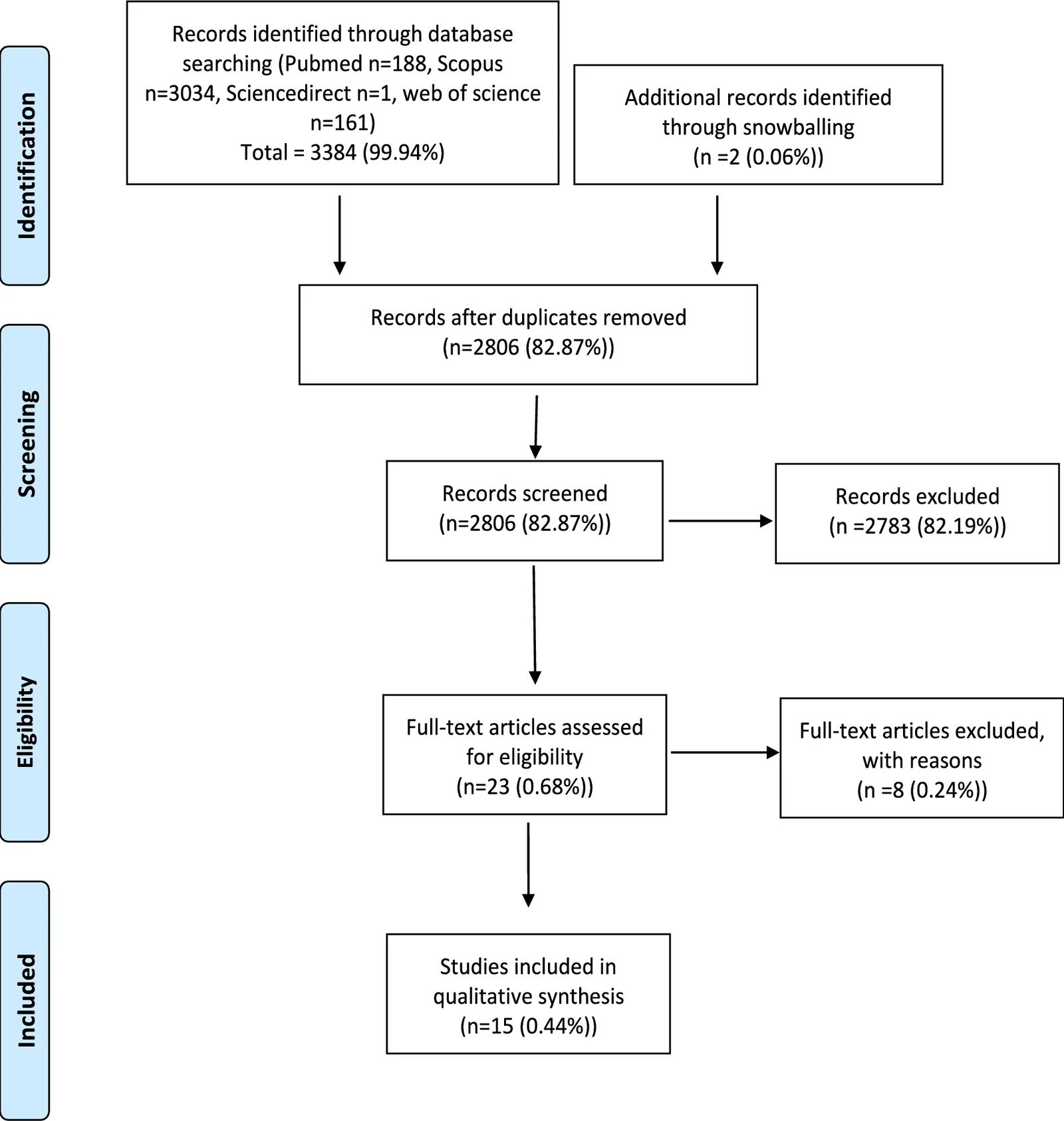
Preferred Reporting Items for Systematic Reviews and Meta-Analyses (PRISMA) flowchart

Study selection was carried out using Rayyan [32] and performed in three phases through reading titles, abstracts, and full texts: identification, screening, and eligibility as shown in Fig. 1.

#### Inclusion criteria

Primary research articles written in English and published in peer-reviewed journals, with a focus on known mosquito vectors including all species belonging to the genera *Culex*, *Aedes,* and *Anopheles* and others, and arboviruses known to cause infections in vertebrates. Only in vivo studies were accepted and studies had to specifically report on the effects of the ISV on the arbovirus co-infection or transmission dynamics within the mosquito for instance, reduced viral load, change in transmission rates, and immune response. For this study, in vivo studies experiments are conducted using live and intact mosquitoes.

#### Exclusion criteria

Any reviews and editorials that do not contain the main primary data. All research articles that use cell lines instead of actual mosquitoes as vectors, these are in vitro studies. For this study, in vitro studies are experiments conducted in an artificial environment outside the mosquitoes, usually in cell culture. Studies that only reported the isolation and characterization of the ISVs without investigating their interactions with mosquitoes and arboviruses were also excluded.

The risk of bias and overall quality of the included studies was assessed using the Mixed Methods Appraisal Tool (MMAT) [33]; based on three key elements: (1) the appropriateness of the study design in relation to the review question, (2) the quality of the execution of the study methodology, and (3) the relevance of the study to the review objectives. Differences of opinion over the inclusion of an article were resolved through discussion between two authors: AM and NM.

### Data extraction

Data from relevant studies were extracted independently by AM and were reviewed independently by NM. Data extraction forms were developed from the template provided by the Joanna Briggs Institute (JBI) guidelines [34]. Data were extracted from the articles into a Microsoft Excel file and organized under the relevant headings by AM. Once the process was complete, the data were again reviewed by NM and displayed in tabular form. The extracted data included author, year, mosquito species, ISV/family/route of infection, geographic origin of the strain, arbovirus(es) being investigated, arbovirus infection route, association of ISV with arbovirus-effects on infection, replication and transmission, and methodology used. Disagreements between individual’s judgements were resolved by revising the study and reaching a consensus agreement.

## Results

### Selection of sources of evidence

The database search produced a total of 3386 articles, including two additional articles identified through snowballing, of which 580 were removed because they were duplicates. Based on the inclusion criteria 2783 articles were further excluded from the study. A total of 23 articles proceeded to full text screening, of which eight articles were excluded, because they were in vitro studies. A total of 15 articles were included in this review (Fig. 1).

### Risk assessment

Individual studies were assessed for risk of bias (Table 1). For the “representative sample size” criterion, studies with a sample size ≥ 100 were considered to have adequate population representation; however, this threshold is arbitrary and constitutes a limitation of the risk-of-bias assessment. Each study was evaluated against the MMAT Quantitative descriptive criteria and methodological quality was categorized as high when at least 80% of the criteria was met and low when less than 50% of criteria were satisfied. Overall, all 15 studies were categorized as high quality, they clearly defined their research questions and employed appropriate methods, statistical analysis, and sampling strategies. However, the representativeness of the sample size varied, for most studies it was not clear on the sample size used, while some studies met the predefined threshold of ≥ 100 [20, 22, 24, 35, 36]. Sample size is an important parameter in estimating vector competence; the use of small or non-representative samples may overestimate or underestimate inhibitory or enhancing effects due to reduced population size. All 15 studies were included for the review as they had minimum risk of bias [20, 22, 24, 35, 36].

**Table 1:** Risk of bias assessment

Category of study Designs	Methodological quality criteria	Article references with responses														
		[24]	[22]	[20]	[18]	[21]	[17]	[37]	[35]	[36]	[25]	[26]	[27]	[28]	[30]	[29]
Screening questions	Are there clear research questions?	Yes	Yes	Yes	Yes	Yes	Yes	Yes	Yes	Yes	Yes	Yes	Yes	Yes	Yes	Yes
	Does the data collected allow me to address the research questions?	Yes	Yes	Yes	Yes	Yes	Yes	Yes	Yes	Yes	Yes	Yes	Yes	Yes	Yes	Yes
Quantitative descriptive	Is the sampling strategy relevant to address the research question?	Yes	Yes	Yes	Yes	Yes	Yes	Yes	Yes	Yes	Yes	Yes	Yes	Yes	Yes	Yes
	Is the representative sample size appropriate for the study? *	Yes	Yes	Yes	UC	UC	UC	UC	Yes	Yes	Yes	UC	UC	UC	UC	UC
	Are the methods appropriate?	Yes	Yes	Yes	Yes	Yes	Yes	Yes	Yes	Yes	Yes	Yes	Yes	Yes	Yes	Yes
	Is the risk of non-response bias low?	Yes	Yes	Yes	Yes	Yes	Yes	Yes	Yes	Yes	Yes	Yes	Yes	Yes	Yes	Yes
	Is statistical analysis appropriate to answer the research question?	Yes	Yes	Yes	Yes	Yes	Yes	Yes	Yes	Yes	Yes	Yes	Yes	Yes	Yes	Yes

UC, Unclear

^*^Representative size sample was considered appropriate if ≥ 100 mosquitoes

### Reports on the effects of interactions between mosquitoes, ISVs, and arboviruses on replication and transmission of arboviruses and infection of mosquito cells in vivo

Reports were available for 11 ISVs including cell-fusing agent virus (CFAV), Nhumurim virus (NHUV), Palm Creek virus (PCV), *Culex flavivirus* (CxFV), Phasi Charoen-like virus (PCLV), Humaita-Tubiacanga virus (HTV), Espirito Santo virus (ESV), Yichang virus (YCV), *Anopheles gambiae* densovirus (AgDNV), *Aedes aegypti* densovirus (AaeDV), and Eilat virus (EILV). A summary of the data retrieved on the influence of ISVs on transmission and replication of co-infecting arboviruses, such as DENV, ZIKV, WNV, RVFV, MAYV, and CHIKV, in *Culex* and *Aedes* mosquito species, is shown in Table 2. Briefly, 8/15 studies reported on ISFs, the most characterized ISVs probably due to their genetic homology to significant pathogens such as WNV, DENV, and ZIKV. The remaining studies reported other ISVs from the viral families Phenuiviridae*, Birnaviridae, Mesoniviridae, Parvoviridae, Togaviridae,* and the unclassified (HTV). The effect of ISVs on transmission, replication, and infection of arboviruses was variable. Five studies reported no significant impact on co-infected mosquitoes [18, 20, 22, 29, 36]. Overall there was no significant change in replication, in infection or dissemination rates and the transmission efficiency remained the same. In contrast, inhibitory effects on one or more vector competence parameters, replication, transmission, and infection rate of arboviruses were described in nine studies [17, 18, 20, 25–27, 30, 35, 37]. Effects observed included reduced arbovirus replication in mosquito cells or tissues, lower infection rates in the mosquito midgut, reduced dissemination from midgut to other tissues, and lower transmission potential through saliva [17, 18, 20, 25–27, 30, 35, 37]. Three studies reported an increase in vector competence parameters [21, 24, 29], higher infection rates, increased viral titers, and increased transmission potential, perhaps due to immune suppression and altered host metabolism.

**Table 2:** Summary of reports describing in vivo co-infections of ISVs and arboviruses

ISV species	ISV family	Mosquito species/ cell line where the ISV was isolated for the study	ISV route of infection	Arbovirus(es)	Arbovirus infection route	Mosquito species analyzed	Overall impact on vector competence parameters			Timing of effects	Method(s) used to detect and quantify arbovirus	References
							Transmission/ dissemination	Infection	Replication			
CFAV	*Orthoflaviviridae*	*Ae. aegypti* mosquitoes (colony) from Thailand; isolate obtained using *Ae. albopictus* C6/36 cells	Artificial-intrathoracic	DENV-1/ZIKV	Oral blood feeding	*Ae. aegypti*	Reduced(potentially)	–	Reduced	Reduced dissemination during early infection	RT–qPCR RT-PCR	[37]
HTV	*–*	*Ae. aegypt*i mosquitoes from Brazil	Natural	DENV-1/ZIKV	Oral blood feeding	*Ae. aegypti*	Increased (ZIKV)	Increased (DENV)	Increased (DENV/ZIKV)	Persistent	RT–qPCR RT-PCR	[24]
PCLV	*Phenuiviridae*	*Ae. aegypt*i mosquitoes from Brazil	Natural	DENV-1/ZIKV	Oral blood feeding	*Ae. aegypti*	Increased (ZIKV)	Increased (DENV)	Increased (DENV/ZIKV)	Persistent	RT–qPCR RT-PCR	(24)
NHUV	*Orthoflaviviridae*	*Cx. chidesteri* mosquitoes from Brazil; *Ae. albopictus* C6/36cells	Artificial-intrathoracic	ZIKV	Oral blood feeding & Artificial-intrathoracic	*Ae. aegypti*	Reduced (Co-infection)	Reduced	Reduced	Persistent	RT–qPCR Plaque titration	[35]
NHUV	*Orthoflaviviridae*	*Cx. chidesteri* mosquitoes from Brazil; *Ae. albopictus* C6/36 and C7-10 cells	Artificial-intrathoracic	WNV	Artificial-intrathoracic	*Cx. pipiens*	No significant difference	No significant difference	–	–	RT–PCR IFA	[18]
						*Cx. quinquefasciatus*	Reduced	No significant difference	–	–	RT–PCR IFA	
CxFV	*Orthoflaviviridae*	*Cx. quinquefasciatus* from Guatemala; *Ae. albopictus* C6/36 cells	Artificial-intrathoracic	WNV	Artificial-intrathoracic	*Cx. quinquefasciatus*	Increased	No significant difference	No significant difference	Significant differences in WNV titers was seen at 0 dpi (lower) and 4 dpi (higher) disappeared by 8 and 10 dpi	RT–qPCR Plaque titration	[21]
ESV	*Birnaviridae*	*Ae. albopictus C6/36 cells*	Artificial-larval immersion in viral solution and sugar solution containing the virus	DENV-2	Oral blood feeding	*Ae. aegypti*	Reduced	No significant difference	Reduced	–	RT-qPCR Plaque titration	[26]
CxFV	*Orthoflaviviridae*	*Cx. pipiens* mosquitoes from the United States of America; Ae*. albopictus* C6/36 cells	Natural	WNV	Oral blood feeding	*Cx. Pipiens*	No significant difference	No significant difference	Reduced			
CxFV	*Orthoflaviviridae*	*Cx. pipiens* mosquitoes from Spain; *Ae. albopictus* C6/36 cells	Artificial-intrathoracic	RVFV	Oral blood feeding	*Cx. Pipiens*	No significant difference	No significant difference	No significant difference			
PCV	*Orthoflaviviridae*	*Cq. xanthogaster* mosquitoes from Australia; *Ae. albopictus* C6/36 cells	Artificial-intrathoracic	WNV	Oral blood feeding	*Cx. annulirostris*	Reduced	Reduced	Inconclusive			
					Artificial-intrathoracic	*Cx. annulirostris, Ae. aegypti; Ae. vigilax*	No significant difference	No significant difference	Inconclusive			
PCV	*Orthoflaviviridae*	*Cq. xanthogaster* mosquitoes from Australia; *Ae. albopictus* C6/36 cells	Artificial-intrathoracic	ZIKV/CHIKV	Oral blood feeding	*Ae. aegypti*; *Ae. albopictus*	No significant difference	No significant difference	–			
ESV	*Birnaviridae*	*Ae. albopictus C6/36 cells*	Artificial–larval immersion in viral solution and sugar solution containing the virus	DENV-2	Oral blood feeding	*Ae. aegypti*	Reduced	No significant difference	Reduced			
YCV	*Mesoniviridae*	*Ae. albopictus C6/36 cells*	Oral feeding	DENV-2	Oral feeding	*Ae. albopictus*	Reduced	Reduced	No significant difference			
AgDNV	*Parvoviridae*	*Anopheles gambiae cell line Sua5B*	Artificial-intrathoracic	MAYV	Oral blood feeding	*An. gambiae*	Reduced	No significant difference	Reduced			
EILV	*Togaviridae*	*Anopheles coustani*	Artificial-intrathoracic	CHIKV	Oral blood feeding	*Ae. aegypti*	Reduced	No significant difference	No significant difference			
EILV	*Togaviridae*	EILV cDNA clone	Oral blood feeding	WNV	Oral blood feeding	*Cx. tarsalis*	No significant difference	No significant difference	No significant difference (Increased)			

CFAV-cell-fusing agent virus NHUV-Nhumurim virus PCV-Palm Creek virus CxFV-*Culex flavivirus* PCLV-Phasi Charoen-like virus HTV-Humaita-Tubiacanga virus ESV-Espirito Santo virus YCV- Yichang virus AgDNV-Anopheles gambiae densovirus AaeDV-Aedes aegypti densovirus EILV-Eilat virus DENV-dengue virus CHIKV-chikungunya virus WNV-West Nile virus ZIKV-Zika virus RVFV-Rift valley fever virus MAYV- Mayaro virus RT-qPCR- reverse transcriptase quantitative Polymerase Chain Reaction RT-PCR-reverse transcriptase- polymerase chain reaction IFA-Immunofluorescence Assay IHC-Immunohistochemistry

Included studies indicate that ISVs were predominantly (10/15 studies) artificially introduced into mosquitoes using intrathoracic inoculation, while natural infection was less common. Intrathoracic inoculation was dominated by inhibitory effects, while enhancing effects were observed in natural infection. Semi-quantitative synthesis indicates that inhibitory (approximately 40%) or neutral (approximately 40%) effects on arbovirus infection dynamics were commonly observed while enhancing effects being less common (less than 20%) reported. Inhibitory effects were more frequently observed for replication and dissemination than in infection, while neutral effects (no significant effects) were frequently reported for infection. Most studies used *Aedes* species as their host and both enhancement and inhibition were reported in *Aedes*, while *Culex* and *Anopheles* mosquitoes showed predominantly neutral or inhibitory outcomes. Enhancement effects on arboviruses were only observed on flaviviruses, particularly DENV and ZIKV, whereas alphaviruses were mostly unaffected or suppressed.

Notably, several studies reported temporal dynamic effects, with transient inhibition of arbovirus replication or dissemination observed on or before 7 dpi, followed by attenuation or loss of these differences after 14 dpi. Semi-quantitatively, early inhibitory effects were observed in approximately 50% of the studies, while later time points were more frequently characterized by no significant differences.

## Discussion

Insect-specific viruses are a distinct group of viruses that naturally infect mosquitoes and are maintained primarily within mosquito populations through vertical transmission and cannot replicate in vertebrates [38]. Their ability to interfere with or alter the replication, infection, and transmission rates of arboviruses of medical significance has sparked interest in their possible use as biological control agents. In addition to their efficient transmission within mosquito populations, ISVs are maintained via vertical and horizontal transmission, which enables their sustained presence within an ecosystem [39]. This review looked at the existing evidence on the impact of ISVs on replication, transmission, and infection of arboviruses in mosquitoes.

Interactions between ISVs and arboviruses in mosquitoes are variable, with effects that can either inhibit or enhance vector competence, including replication, infection, and transmission, or have no impact. It is acknowledged that the underlying mechanisms are unknown, and a direct causal link needs to be fully investigated. While there appears to be some influence on co-infections that warrants further investigation, the included studies primarily report phenotypic outcomes and do not directly investigate the underlying mechanisms. The relationships between ISVs, arboviruses, and their mosquito hosts are complex and several factors determine the outcomes, including environment, evolutionary history of the mosquito species and the associated viruses, viral relatedness, arboviruses vs ISVs, mosquitoes’ immune responses, and experimental design [25, 37, 39].

One of the major factors that determine the outcome of the interactions is whether the ISV–mosquito association is naturally occurring or artificially generated in the laboratory. When ISVs circulate naturally in wild mosquito populations, they become a product of long-term co-evolution and tend to establish a mutual relationship with their hosts [37, 40]. In natural setups, ISVs can enhance arbovirus transmission, as observed for HTV and PCV in *Ae aegypti* [24]. Moreover, mosquitoes in the wild frequently harbor multiple ISVs simultaneously, creating a complex virome in which synergistic or antagonistic interactions among symbionts may collectively shape vector competence [24, 41, 42]. On the other hand, artificial ISV infections, especially those introduced via intrathoracic injection, more frequently result in suppression of arbovirus replication [20, 25, 35, 37]. The suppression of arboviral replication may be due to disrupted tissue tropism, altered immune activation, and/or non-ecological viral loads that do not occur under natural conditions. However, these mechanisms were not directly investigated in the included studies and remain speculative. Importantly, intrathoracic injection bypasses natural anatomical barriers such as the midgut and salivary glands and may therefore overestimate the inhibitory effects observed under experimental conditions, limiting their direct relevance to biocontrol applications in natural settings [39, 43].

Another factor that influences the interaction outcome is the phylogenetic relationship between ISVs and arboviruses. When ISVs and co-infecting arboviruses belong to the same order and genus, such as flaviviruses or alphaviruses, competition for shared host factors may lead to superinfection exclusion [44]. Superinfection exclusion is when a pre-existing viral infection blocks the replication of a second, closely related virus by competing for cellular resources or replication sites. In agreement, negative associations were observed for homologous in vivo artificial associations such as CFAV-DENV/ZIKV, NHUV-ZIKV, EILV-CHIKV, and PCV-WNV [20, 25, 35, 45]. In a separate in vitro study (though not included in this review) it was demonstrated that NHUV inhibited the replication of flaviviruses such as Saint Louis encephalitis virus, Japanese encephalitis virus, and WNV in mosquito cell lines [46]. Interestingly some studies demonstrated early suppression of arboviruses by ISVs most likely because of competition between binding sites and/or receptors; this shows that ISV-mediated interference may be more pronounced during initial infection and is not consistently sustained throughout the transmission process. In the laboratory, WNV replication was suppressed in *Cx. pipiens* naturally infected with CxFV, up to 7 days post infection (dpi) with no significant effects at 14 dpi, moreover no significant effects on arbovirus transmission rate were observed 14 dpi in a naturally infected mosquito colony [17]. These results suggest a competitive interaction between CxFV and WNV in the early stages of WNV infection. However, in this study the genetic background of the *Cx. pipiens* used for the negative control differed from the positive making any conclusive findings difficult to determine. Some studies have indicated that ISV and arbovirus interactions are dependent on viral strains and mosquito species [37, 47]; hence, there is a need for more studies to compare the effects of using identical mosquito species.

Most importantly, phylogenetic studies indicate that arboviruses may have evolved from ISVs through the development of dual tissue tropism [50, 51]. On the other hand, some theories state that ISVs from alphaviruses originally had a dual tropism that they lost [52, 53]. The phylogenetic relationships of ISVs and arboviruses are not fully understood and complex, which raises critical considerations such as the possibility of recombination or adaptation influencing arbovirus emergence, such processes remain speculative and were not addressed in the studies included in this review.

In addition to superinfection exclusion mechanism, upregulation of antiviral responses, including RNA interference (RNAi) pathways (siRNA and piRNA) and Janus kinase (Jak)-signal transducer and activator of transcription (STAT) (Jak-STAT) activation via Vago have been found to modulate associations between ISVs and arboviruses [39]. Several studies indicate that ISVs may prime antiviral pathways, including RNA interference, thereby restricting subsequent arbovirus infection, although direct evidence from the included studies is limited [39, 48]. Conversely, some ISVs alter host gene expression to favor arbovirus replication. For example, HTV and PCV prevent the downregulation of histone H4, a proviral factor required for efficient dengue and Zika virus replication, thereby enhancing arbovirus replication and transmission [24]. In addition, EILV initially enhanced WNV NY99 titers at 3 dpi, though there was no significant difference at the end of infection period, 7 dpi [29]. This transient enhancement may be driven by strain-specific differences in resource exploitation or interaction with the host immune response (e.g., RNAi) that temporarily allow the NY99 strain to overcome initial EILV-mediated exclusion, although this remains speculative. Another explanation could be that NY99 “piggybacks” and infects more cells or cell types with the help of EILV. This strategy is used by the human immunodeficiency virus during co-infection with viruses such as Epstein–Barr virus to expand its tissue tropism in its host [49]. These findings demonstrate that ISVs can act as regulators of host susceptibility rather than merely as viral competitors.

Other factors that contribute to the variations in association outcomes include the environment, route of infection, timing, and quantities of ISVs. For instance, while co-infection with PCV effectively inhibited WNV and Murray Valley encephalitis virus (MVEV) in vitro and inhibited WNV in Culex mosquitoes, it showed little to no effect on ZIKV or CHIKV in *Ae*. *aegypti* and *Ae*. *albopictus* [22]. During natural infection of mosquitoes with viruses, virus cross specific anatomical barriers in the mosquito, including the midgut and salivary glands, to be transmitted to vertebrate hosts and/or complete their life cycle [44]. Artificial infection enables introduction of ISVs into mosquito species that may not be permissive under natural conditions. However, ISVs introduced artificially bypass natural barriers and establish atypical tissue distributions, potentially exaggerating their inhibitory effects [20, 22]. In contrast, naturally acquired ISVs are obtained through oral routes and can also be transmitted vertically and display tissue tropism shaped by long-term host–virus co-adaptation [24, 39]. Therefore, experimental infection route is a critical methodological variable influencing observed ISV–arbovirus interactions. PCV demonstrated contradictory results depending on the route of infection. Some studies employed intrathoracic injection to introduce ISVs into mosquitoes, but intrathoracic injections may not accurately represent natural infection dynamics or the conditions under which ISV-based biocontrol strategies would operate and findings from such studies should be interpreted with caution. Moreover, timing and the ratio of ISV to arbovirus exposure determine the interaction outcomes. High ISV loads established prior to arbovirus challenge are more likely to interfere with arbovirus replication, whereas low-level or late ISV infections may have minimal or even facilitating effects [18, 23].Reference: Reference [32] is given in list but not cited in text. Please cite in text or delete from listI have cited reference 32 in textKindly check and confirm the placement of [Disclaimer] section was appropriate. Otherwise advise us on how to proceed.I have checked, it is fin

Future research should concentrate on clarifying the molecular mechanisms behind these interactions, standardizing experimental conditions across vector species, and examining the ecological fitness and transmission potential of candidate ISVs in semi-field or field environments. Combining phylogenetic studies, molecular virology, vector ecology, and genomics will be essential for determining if ISVs can be effectively and safely used as part of integrated vector management strategies.References: As References [30 and 39] and [22 and 35] are same, we have deleted the duplicate reference and renumbered accordingly. Please check and confirm.Checked, its ok

## Conclusions

The current evidence indicates that interactions between ISVs and arboviruses are complex, strain-dependent, and context specific. Certain ISVs show potential as biological inhibitors of arboviruses, while others facilitate viral replication and exhibit neutral effects. Therefore, ISVs should be regarded as modulators of arboviral transmission dynamics, with effects that are dependent on specific virus-vector combinations, rather than universal biocontrol agents. As such, there is a need for comprehensive in vivo studies and ecological assessments before ISVs can be considered viable tools for arbovirus control in natural mosquito populations.

## Supplementary Information

Additional file 1.

Additional file 2.

## Data Availability

Data supporting the main conclusions of this study are included in the manuscript.
