# Supplementary material for: Effects of insect-specific viruses on transmission, infection, and replication of arboviruses in co-infected mosquitoes: a systematic review
Source: Parasit Vectors. 2026 Jun 23;19:381. doi: 10.1186/s13071-026-07495-x (PMC13543357; doi:10.1186/s13071-026-07495-x)
Supplement: Supplementary file 1 [file 13071_2026_7495_MOESM2_ESM.docx]

**Effects of insect-specific viruses on transmission, infection and replication of arboviruses in co-infected mosquitoes: A systematic review**

Angela Sibanda-Makuvise ^1,2^ Nosipho Masoto^1^ Gert Ignatius du Preez Terblanche^1^ Puleng Ralepeli^1^ Jolene Botha^1^ Felicity J. Burt ^1,3^

Supplement S1: Search strategy and search terms

1. Pubmed

Search: **( insect-specific flavivir* OR ISF OR Insect-specific vir* OR ISV ) AND ( biocontrol OR replication OR transmission OR inhibition ) AND arbovir* AND mosquit***

(("insect specific"[All Fields] AND "flavivir*"[All Fields]) OR "ISF"[All Fields] OR ("insect specific"[All Fields] AND "vir"[All Fields]) OR "ISV"[All Fields]) AND ("biocontrol"[All Fields] OR "biocontrolling"[All Fields] OR "biocontrols"[All Fields] OR ("dna replication"[MeSH Terms] OR ("dna"[All Fields] AND "replication"[All Fields]) OR "dna replication"[All Fields] OR "replication"[All Fields] OR "replicability"[All Fields] OR "replicable"[All Fields] OR "replicate"[All Fields] OR "replicated"[All Fields] OR "replicates"[All Fields] OR "replicating"[All Fields] OR "replicational"[All Fields] OR "replications"[All Fields] OR "replicative"[All Fields] OR "replicatively"[All Fields] OR "replicator"[All Fields] OR "replicators"[All Fields]) OR ("transmissability"[All Fields] OR "transmissable"[All Fields] OR "transmissibilities"[All Fields] OR "transmissibility"[All Fields] OR "transmissible"[All Fields] OR "transmissibles"[All Fields] OR "transmission"[MeSH Subheading] OR "transmission"[All Fields] OR "transmissions"[All Fields]) OR ("inhibit"[All Fields] OR "inhibitable"[All Fields] OR "inhibite"[All Fields] OR "inhibited"[All Fields] OR "inhibites"[All Fields] OR "inhibiting"[All Fields] OR "inhibition, psychological"[MeSH Terms] OR ("inhibition"[All Fields] AND "psychological"[All Fields]) OR "psychological inhibition"[All Fields] OR "inhibition"[All Fields] OR "inhibitions"[All Fields] OR "inhibitive"[All Fields] OR "inhibits"[All Fields])) AND "arbovir*"[All Fields] AND "mosquit*"[All Fields]

2. Scopus and Sciencedirect

ALL ( ( insect-specific flavivir* OR ISF OR Insect-specific vir* OR ISV ) AND ( biocontrol OR replication OR transmission OR inhibition ) AND arbovir* AND mosquit* )

3. Web of Science

( insect-specific flavivir* OR ISF OR Insect-specific vir* OR ISV ) AND ( biocontrol OR replication OR transmission OR inhibition ) AND arbovir* AND mosquit* (All Fields)

<https://www.webofscience.com/wos/woscc/summary/b2a9eafd-7bc7-4915-a1ce-745fc10c7657-01b18cd5fa/relevance/1>
