# Supplementary material for: Effects of insect-specific viruses on transmission, infection, and replication of arboviruses in co-infected mosquitoes: a systematic review
Source: Parasit Vectors. 2026 Jun 23;19:381. doi: 10.1186/s13071-026-07495-x (PMC13543357; doi:10.1186/s13071-026-07495-x)
Supplement: Supplementary file 2 [file 13071_2026_7495_MOESM1_ESM.doc]

**PRISMA 2009 Flow Diagram**

**Screening**

**Included**

**Eligibility**

**Identification**

Records identified through database searching (Pubmed n=188, Scopus n=3034, Sciencedirect n=1, web of science n=161)
Total = 3384 (99.94%)

Additional records identified through snowballing
(n =2 (0.06%))

Records after duplicates removed
(n=2806 (82.87%))

Records screened
(n=2806 (82.87%))

Records excluded
(n =2783 (82.19%))

Full-text articles assessed for eligibility
(n=23 (0.68%))

Full-text articles excluded, with reasons
(n =8 (0.24%))

Studies included in qualitative synthesis
(n=15 (0.44%))
